# Reducing Energy Consumption and Health Hazards of Electric Liquid Mosquito Repellents through TinyML

**DOI:** 10.3390/s22176421

**Published:** 2022-08-25

**Authors:** Inyeop Choi, Hyogon Kim

**Affiliations:** Department of Computer Science and Engineering, Korea University, Anam-Dong, Sungbuk-Gu, Seoul 02841, Korea

**Keywords:** TinyML, electric liquid mosquito repellent, prallethrine, convolutional neural network (CNN), embedded AI, health, energy saving

## Abstract

Two problems arise when using commercially available electric liquid mosquito repellents. First, prallethrine, the main component of the liquid repellent, can have an adverse effect on the human body with extended exposure. Second, electricity is wasted when no mosquitoes are present. To solve these problems, a TinyML-oriented mosquito sound classification model is developed and integrated with a commercial electric liquid repellent device. Based on a convolutional neural network (CNN), the classification model can control the prallethrine vaporizer to turn on only when there are mosquitoes. As a consequence, the repellent user can avoid inhaling unnecessarily large amounts of the chemical, with the added benefit of dramatically reduced energy consumption by the repellent device.

## 1. Introduction

According to ResearchAndMarkets.com [1], the global market size for mosquito repellents is expected to reach 9 billion US dollars by 2026 up from 6.9 billion in 2021 at a compound annual growth rate of 5.6%. Mosquito repellent product types include coils, liquid vaporizers, aerosol sprays, mats, creams and oils. Among them, electric mosquito repellents account for 30% of the market [2]. In 2018, this was 29.7% [3] in the US market and 28.71% [4] in Korea. Recently, the liquid vaporizer type has become more popular. There is a dearth of statistics on the mosquito repellent market size as far as the liquid vaporizer type is concerned.

However, according to Yonhap News in Korea, 10 million units of HomeMat Electric Mosquito Repellent, which account for 50% of the Korean electric vaporizer market, have been sold in Korea over 20 years [5]. Considering the typical multi-year lifetime of the device, this implies that several million electric mosquito repellents are being used in Korean households. Furthermore, in the Korean market, the liquid vaporizer type, which can be used without changing the liquid cartridge for as long as one month, is further increasing in use, compared with the second most popular mat type that requires changing the mat after 14 h.

There are two problems with the chemical diffuser-type repellents, such as the liquid vaporizer. First, the user has to inhale the vaporized chemical over an extended period of time, in particular while sleeping overnight, regardless of whether mosquitoes are present or not. The ingredients commonly used in mosquito repellents are prallethrine and allethrin, which are members of the pyrethroid class of insecticides—the former being the main ingredient [6]. If this ingredient is used in a closed room with poor ventilation, side-effects, such as rhinitis, asthma, lethargy, sneezing, headache, tinnitus and nausea, may appear.

In addition, Quirós-Alcalá [7] published a study finding that American children exposed to pyrethroid insecticides had higher rates of learning disabilities and attention-deficit/hyperactivity disorder (ADHD). The second problem is that, if the mosquito is not in the room, or even after the mosquito is killed by the chemical, the current commercial devices keep consuming electricity unnecessarily. They are simply not designed to determine if mosquitoes are present. The decision to turn on the device is up to the user who in most cases cannot determine if and where mosquitoes are present.

By nature, mosquitoes hide in places that people cannot easily see and appear only when people do not actively chase them. Thus, to avoid mosquito bites, people tend to keep the mosquito repellent on continuously, especially during night. Most electric mosquito repellent devices are known to consume approximately 5 W of power. If used for 8 h a day, the energy consumption per day will be 40 Wh. According to Yonhap News  [5], 10 million liquid devices have been sold in Korea in 20 years. If we assume that one million of them are being actively used in three summer months, as much as 3.6 GWh of electricity would be consumed per year by these devices in Korea alone.

Despite the health and energy consumption issues, the repellents have not been modified to address them mainly due to the low price of the devices and the chemicals used in them. For instance, even the most expensive vaporizer is less than 30 US dollars. However, we argue that, due to the rapid progress of TinyML technology, it has become feasible both technically and economically to introduce intelligence to accurately determine the presence of mosquitoes into electric liquid repellent devices.

This paper demonstrates this by implementing a convolutional neural network (CNN) model that identifies mosquito sounds and vaporizer control logic in a repellent circuit using TinyML. The energy saving factor of the prototype compared to the existing commercial product can reach 1/8 when mosquitoes are present and 1/227 when they are not. In addition, it can shorten the vaporization time by more than 4 h and reduce the use of repellent liquid to much less than 1 mL.

The false positive rate (FPR) and false negative rate (FNR) in the CNN classifier are also low. The FPR is as low as 0.2% for each classification operation, which is further reduced by using a false positive suppression technique applied over multiple classifications. The FNR depends on the duration of a given mosquito sound. For example, if a mosquito sound lasts 10 s, the FNR can be as large as 70% at the distance of 100 cm; however, in the most critical (i.e., biting) distances of 50 cm or less, the FNR is 0%.

Our prototype demonstrates an example where TinyML can be employed to solve a real-life problem, such as the health hazards and energy consumption in the electric mosquito repellents. Due to the difficulty of controlled experiments with live mosquitoes, we used a database of mosquito sound to train and test the developed prototype. Although not definitive proof of the expected performance, the test results suggest that the proposed system can operate at reduced electric power and at high accuracy.

The rest of the paper is organized as follows. Section 2 briefly lists the works that illuminate the capabilities and potentials of TinyML. This section also enumerates the efforts to apply TinyML in various application areas ranging from healthcare to farming to surveillance to traffic control system among others. Section 3 develops a convolutional neural network (CNN) model for mosquito sound classification to be ported to the electric mosquito repellent system as the target.

After the model is trained to have high accuracy, Section 4 discusses how the intelligence is ported to a commercial liquid electric mosquito repellent device for which TinyML is leveraged. Section 5 evaluates the prototype mosquito repellent system that is equipped with the developed intelligence in controlling the evaporation activity to reduce the vaporized chemical and the consumed power. Finally, Section 6 concludes the paper.

## 2. Related Work

Although artificial intelligence (AI) can help solve many real-world problems, it is challenging to imbue it into low-end embedded systems. Since embedded systems perform only specific tasks, the computing resources are limited. Microcontrollers installed in low-power systems, such as home appliances and IoT devices, usually have hundreds of KBytes of SRAM and less than 1 MB of Flash memory. Fortunately, TinyML is an enabling technology to adapt AI on resource-limited systems. Dutta [8] discussed changes when Internet-of-Things (IoT) devices meet the TinyML technology.

The traditional approach of IoT devices is to rely on the cloud to receive machine-learning (ML) services for data generated by IoT devices. However, by using TinyML for the microcontroller unit (MCU) of IoT devices connected to the sensor, this can execute the ML algorithm locally, reducing network overload, latency, communication costs with the server and potential crashes during communication and energy generated during communication. It improves the security, privacy and reliability. Ray [9] introduced the latest hardware platform, deep learning framework and library to support TinyML. It also provides the latest information on the frameworks for TinyML that are being developed by companies and research groups.

Doyu [10] proposed an ecosystem surrounding TinyML called TinyMLaaS, a SaaS—a subscription software. In order to hide the heterogeneity of ML support chips and compilers and to support them in an “as a service” manner, they proposed to build a higher-level abstraction of TinyML software to be hardware and software agnostic. Soro [11] stated that TinyML will enable the development of new types of ubiquitous, lightweight and low-cost surveillance, monitoring and identification applications. TinyML is particularly well-suited for applications that require simple, categorized answers and can be applied to any application with these characteristics.

There have been several works that applied TinyML to solve real-life problems. These problems range across healthcare, surveillance, embedded security, industrial monitoring, autonomous system, augmented/virtual reality, smart space, etc. TinyML can enable embedded systems to perform complex tasks, such as image processing and voice recognition, that could not be performed before [8]. Alati et al. [12] used TinyML operating in a low-power environment to introduce a machine-learning algorithm for temperature prediction in a greenhouse.

Roshan et al. [13] applied TinyML to a traffic control system (TCS). The traffic signal timing of current TCSs are mostly statically configured; thus, it is not suitable for dealing with probabilistic processes, such as vehicle traffic. Raza et al. [14] applied TinyML to unmanned aerial vehicles (UAVs) whose operation times are limited by power consumption issues.

Advanced decision-making capabilities must be provided in an energy-efficient manner to perform sophisticated missions without significantly limiting the flight time. Ooko [15] mentioned the importance of TinyML application in places, such as Africa, where there is a shortage of funding and infrastructure support for disease monitoring.

The Offline Cholera Detector System could detect waterborne cholera as an offline edge AI using TinyML. TinyML was also used for an environmentally friendly IoT-based precision farming system in Rwanda [15]. The system detects various soil parameters and integrates these with predicted weather information to minimize fertilizer use, determine which crops will grow best under the existing conditions and displays the results on a device or mobile phone.

## 3. Developing a Mosquito Sound Classifier

Due to the difficulty of developing the mosquito detection model directly on the commercial electric liquid repellent device, we take a two-step approach. First, we develop the neural network model on a more powerful PC platform where the development environment is better supported. Second, we port it to the repellent device and connect it to the vaporization control. For the mosquito sound classification, we adopt a convolutional neural network (CNN). To use it on the resource-limited device with only 384 KB of SRAM and 1 MB Flash memory, the size of the neural network should be minimized. In this section, we discuss how the model was developed.

### 3.1. Data Set for Model Training on PC

Mosquito sound data for training on the PC platform were collected from humbugdb [16] and YouTube for neural network training. The collected data were split into 1000 one-second-long audio clips. The audio sample rate was set to 16 KHz. In addition to mosquito sound data, ambient living noise data that can be heard in the bedroom or the living room were separately collected. The latter sounds consisted of outside rain, clocks ticking, snoring, human speech, water dripping from the shower, refrigerator motors and TV.

These data were truncated into 1800 one-second-long audio clips. We randomly selected 350 of these to use in the model development. Finally, the background silence noise was synthesized from the collection of white noise and pink noise snippets from the Google data set [17]. These were provided to simulate background noise when training the machine learning on audio clips.

Table 1 summarizes how the sound and noise clips were used in training, validation and testing on the PC platform. All values are the number of sound clips in the given category.

### 3.2. Neural Network Architecture for Mosquito Sound Detection

The neural network detects mosquitoes by distinguishing between mosquito sounds and other sounds. This model is based on CNN. However, since CNNs process images, not sound, the audio clips were first converted to image data. To do this, a Fast Fourier Transform (FFT) was used to transform each audio clip into a spectrogram. In this paper, the FFT algorithm provided by Google was used. After a one-second audio clip was split into 30 ms slices, each slice was converted via FFT into a spectrogram—namely, a 49 × 40 image.

The neural network took this image as input and processed it through three 2D convolution layers and a fully connected (FC) layer. The 2D convolutional layer had eight channels, and each channel had a filter of size 5 × 4. No padding is used, and the stride length was set to 1. The pooling layer used 2 × 2 max pooling. The FC layer has 64 neurons. The output layer had three neurons predicting artificial silence noise (“Silence”), living noise (“Noise”) or mosquito sound (“Mosquito”).

The number of channels in the filter and the number of FC layers were fine-tuned for resource-constrained microcontroller unit (MCU) platforms. Currently, there is only one FC layer, as any increase will result in a larger model size in the number of neurons, which can significantly slow down or, in some cases, even make TinyML applications impossible to run on the MCU. Increasing the number of channels in the filter does not impact the viability of the model as severely; however, it also affects the classification accuracy on the resource-constrained hardware platform.

**Figure 1:**
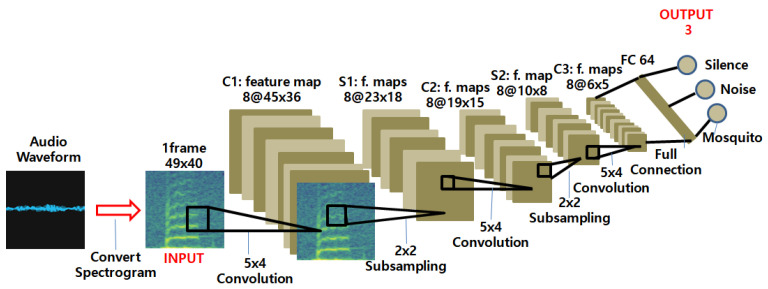
Neural network architecture for mosquito sound detection for the resource-limited hardware platform.

Instead of increasing the model size, the number of hidden layers were increased. Consequently, the number of channels in the filter and the number of FC layers were reduced, while the accuracy was retained. Figure 1 depicts the final CNN architecture for the prototype.

The complexity of a CNN model can be expressed as the number of parameters in each layer. Table 2 shows the complexity of each layer of the mosquito detection neural network model. The last value in the tensor size is the number of channels. For instance, the input layer has only a single channel, for which a 49 × 40 image is given. All convolution and subsampling layers had eight channels. Finally, the fully connected and output layers were vectors. The mosquito detection model used a total of 18,363 parameters to implement the mosquito sound classifier.

### 3.3. Training the Model on a PC Platform

With the architecture shown in Figure 1, we trained it on a PC platform where the computing power is sufficient. The training environment is summarized in Table 3. Once the training was complete, it was optimized and ported to the target platform, i.e., an electric mosquito repellent controller. On the PC platform, a Graphics Processing Unit (GPU) was used to increase the training speed. In order to prevent overfitting on the training data, we used dropouts on three hidden layers (C1, C2 and C3 in Figure 1) with a rate of 0.5 during training. Since Tensorflow 1.15.4 as used in the experiment does not support the latest GPUs, NVIDIA GTX1050 was used, and CUDA10.1 and cuDNN 7.6.5 versions compatible with GTX1050 were used. To compile Tensorflow, we used Bazel, an open source build tool that supports multiple languages and multiple platforms.

**Figure 2:**
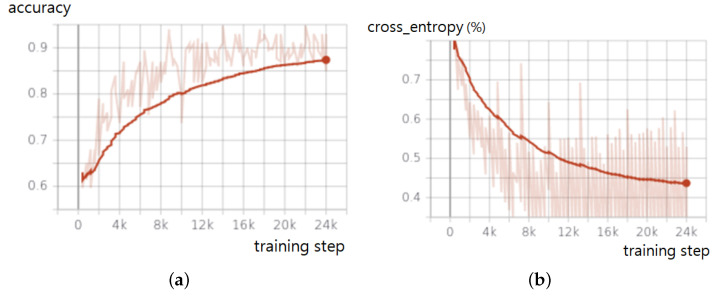
Training accuracy and loss: (**a**) Accuracy. (**b**) Loss measured in cross entropy.

Figure 2 shows the training accuracy and loss of a CNN model using datasets with 1000 mosquito sound clips and 700 noise sound and silence clips. To reduce the model size for the electric liquid repellent device while sacrificing little accuracy, we used the Tensorflow quantization option. If the option was not set, the size of the model became roughly twice that of the quantized version. For model optimization, GradientDescentOptimizer was used, and the sparse_softmax_cross_entropy was used for the loss function. After processing 24,000 randomly selected samples from the data sets, the training reached an accuracy of 88% when the loss was 0.44% (Figure 2).

**Figure 3:**
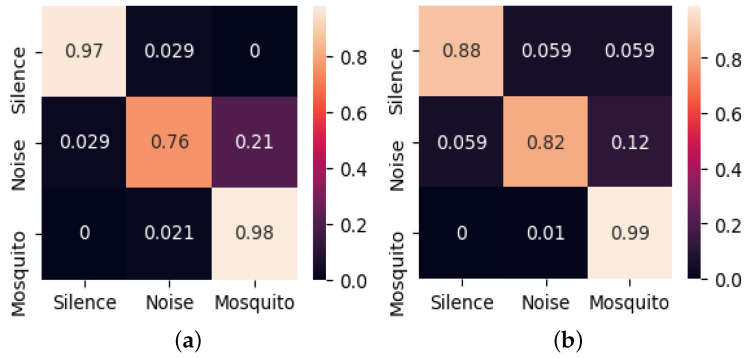
Confusion matrices on PC development platform: (**a**) For validation. (**b**) For testing.

Figure 3 shows the confusion matrices for the validation and testing. The horizontal axis is the ground truth, and the vertical axis is the classification. Recollect that Noise and Silence test samples each take up 21%, whereas mosquito test samples occupy 58%. The validation accuracy was 0.97 × 0.21 + 0.76 × 0.21 + 0.98 × 0.58 = 0.93, and the test accuracy was 0.88 × 0.21 + 0.82 × 0.21 + 0.99 × 0.58 = 0.93. The reason that the validation and the training accuracy are higher than the training accuracy is because of the dropout applied during training.

In the next section, we discuss how the trained CNN model was ported to the microcontroller unit (MCU) on a $15-dollar IoT board [18], which controlled the electric mosquito repellent device. As for the performance of the model on this target platform, it will be presented in Section 5.

## 4. Porting Intelligence to the Electric Mosquito Repellent Device

Below, we first discuss the hardware components of the proposed system—in particular the MCU on the IoT board called SparkFun Edge [18]—that we exploited to run the trained CNN model. We then discuss how the CNN model was embedded on this given hardware platform and the microphone sound preprocessing to use it as input to the CNN model. Finally, we explain how the mosquito detection intelligence was connected to the vaporizer power control.

### 4.1. Hardware

People can determine the presence of mosquitoes by sound. The signature sound gives an almost certain signal that it is a mosquito, especially after the lights are turned off. Therefore, the most effective approach to selectively turning on the mosquito repellent circuit is based on its ability to detect mosquito sounds. When it is determined that there are no mosquitoes, the mosquito repellent is turned off to prevent unnecessary vaporization and energy consumption.

The method proposed in this paper infers the presence of mosquitoes using a convolutional neural network (CNN) model that determines mosquito sounds. Two pieces of additional hardware are essential to implement the inference engine in the repellent device. One is the processor on which the neural network model will run and the other is the microphone needed to hear mosquitoes. Other than them, two auxiliary hardware were also used in our prototype for interfacing the test setup with the commercial electric liquid repellent product.

#### 4.1.1. Microcontroller

The first criterion to be considered when selecting a processor is the price. Electric mosquito repellent devices typically cost between $10 and $30. Thus, the processor should better be cheaper. The second criterion is energy consumption. Since it is impossible to know when mosquitoes will appear, mosquito detection systems must constantly monitor ambient sounds by polling. Significant energy use for continuous monitoring can undermine the value of an intelligent system.

Therefore, the processor used by the mosquito detection system also should consume as little power as possible. Some USB-type devices already have in them a microcontroller unit (MCU) for USB control and button control. MCUs are low-power, low-cost processors. In that case, the developed TinyML application could run on this MCU, and there would be little added cost both in terms of monetary and in terms of energy use.

The MCU used in our prototype was an Appllo3 Blue [19] with a 32-bit ARM Cortex-M4F processor. The CPU clock speed was 48 MHz, and 384 KB SRAM and 1 MB Flash memory were integrated into the MCU. The mosquito detection neural network was stored in the Flash memory and loaded onto SRAM when the system started.

#### 4.1.2. Microphone

Microphones should also be cheap. However, for microphones, sensitivity is also an important criterion. First, it must be able to accept the sound in the 400–500 Hz frequency band emitted by the female mosquito. The narrower the frequency band of the microphone, the cheaper the microphone and the higher the sensitivity. Second, they must have sufficient coverage. For example, if a microphone could only sample the sound of a mosquito within a radius of 10 cm, the sophisticated intelligence implemented in the processor would be of little use. Although the microphone used in this paper does not completely satisfy the above two criteria, a microphone that is as close as possible was selected.

**Figure 4:**
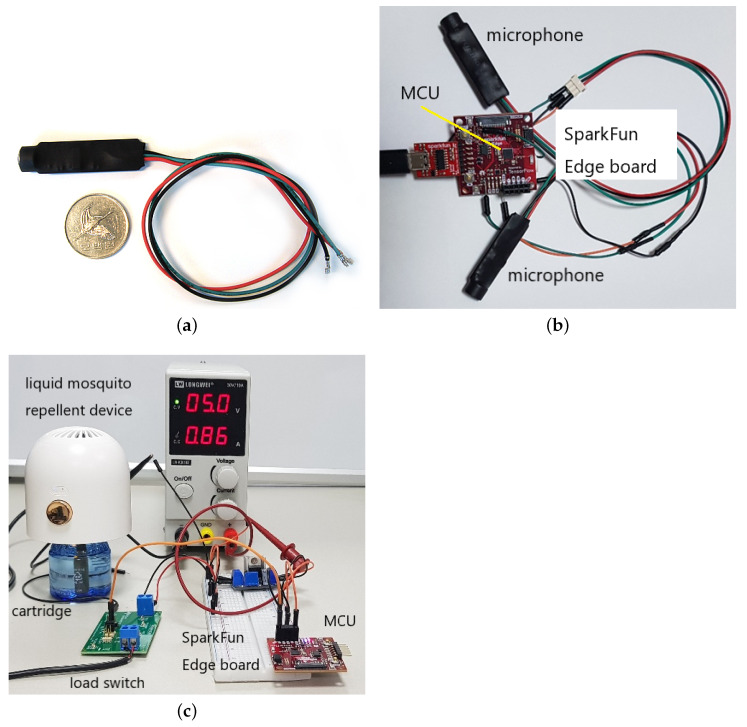
Hardware components in the prototype implementation: (**a**) Voice Band microphone amplifier module (KB100VC) [20] used by the prototype. (**b**) Test board with microphones connected. (**c**) Overall test configuration with a commercial electric liquid mosquito repellent and SparkFun Edge board.

The wingbeat frequency of male mosquitoes is 700–800 Hz and that of female mosquitoes is 400–500 Hz [20]. A microphone designed with a passband of 200 to 4000 Hz was used to pick up mosquito sounds. Figure 4a shows the microphone used for testing.

#### 4.1.3. Test Board

A SparkFun Edge board [18] was used for the prototype. The mosquito detection neural network trained on the Linux PC platform was transferred to the Flash memory in the MCU on the SparkFun Edge board. The SparkFun Edge board connected to a Linux PC via serial communication over a USB-C connection. The SparkFun board did not provide a USB port; thus, we used a USB-to-Serial adapter [21] to connect. The board also supported a general purpose input/output (GPIO) connector for controlling the repellent device. Figure 4b shows the test board with the microphone connected.

#### 4.1.4. Electric Vaporizer Control

A circuit implemented on the test board controlled the vaporization power. The default power state is off and does not power the electric vaporizer. Mosquito-detection neural networks must identify a mosquito sound before turning on the vaporizer. After a certain period of time when there is no longer a positive identification of the mosquito sound, the power is cut back to return to the default state. However, even when the vaporizer is powered off, the neural network detection continuously monitors the ambient sound.

To control the vaporizer’s power supply, the MCU on the SparkFun Edge board uses GPIO. If the test board had been designed from scratch, the GPIO would have been built to directly control the power stage. However, a load switch between the MCU and the repellent device is required to control a commercial USB type repellent device. The MCU’s GPIO controls the power through the load switch. The overall circuit configuration of the intelligent electric mosquito repellent is shown in Figure 4c. Note that the load switch or regulator used in the experimental prototype is only required to control the commercially available mosquito repellent device through the SparkFun Edge board. It is not necessary when designing a board for commercialization of intelligent repellent devices.

### 4.2. TinyML Application

The TinyML application deployed on the target platform discussed above has two main components. The first is the CNN classifier that detects the mosquito sound from the microphone input. The second is the vaporizer power control component that activates the power for the vaporizer based on the output from the classifier and a heuristic logic to reduce the false detection probability. Below, we first discuss the two components of the TinyML application and then how it is packaged to be deployed on the target platform.

#### 4.2.1. Classification by TinyML Application

**Figure 5:**
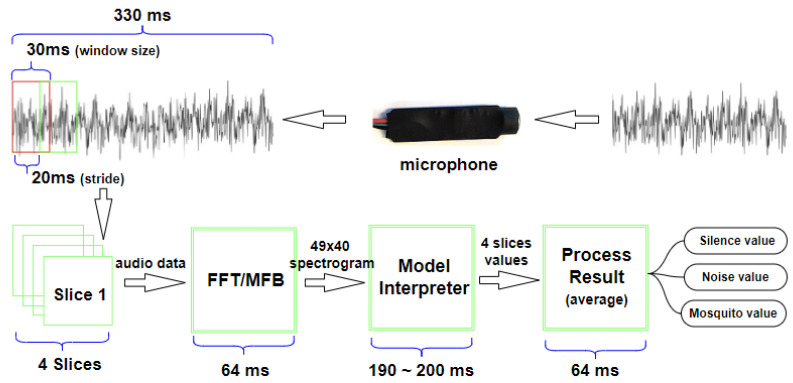
A classification result is produced for every 330 ms audio segment from microphone.

To run the mosquito detection model on the electric liquid repellent device, we first need to process the microphone input for CNN classification. As the classifier has been trained on PC to process 30 ms audio slices, the same format has to be used by the repellent device. Due to the computing power limitation of the MCU on the device, we aim at only three classifications per second. For this, we segment every $L_{seg}=330$ ms of input sound into $L_{slice}=30$ ms slices, among which only the first four slices are processed and averaged for each classification. For continuity, these four slices are overlapped for 10 ms on both ends by using a 20 ms stride. Figure 5 shows the processing chain on the target platform. As can be observed, the processing pipeline takes up to 328 ms to reach the decision. This is why we set the segment length to 330 ms.

In each segment, the first four 30 ms slices are taken with 20 ms stride from the microphone input. Then, each slice goes through Fast Fourier Transform (FFT) and Mel-frequency Filter Bank (MFB) for sound-to-image transformation to use the CNN model in the next step (“Model Interpreter”).

For each 30 ms slice transformed to a 49 × 40 spectrogram image, the CNN classifier outputs a score vector whose sum is 256. This is because the FFT used for our prototype is the 256-Point FFT [22]. The score distribution across the Silence, Noise and Mosquito classes determines the classification at the 30 ms slice granularity. A risk in taking the highest score as the classification result for a given slice may lead to large false positives because the difference between the highest score class and the second highest could be small in many cases. To cope with this problem, we take two approaches.

First, we sum and average the scores for the three classes across the four 30 ms slices in each segment. Second, we apply a high decision threshold for the classification. In our prototype, the threshold θ is set to 210, which is shown to lower the false positive error to below 0.2% for any training data set (see Section 5.2.1). Even if Mosquito has the highest value, it is accepted as a final decision only when the score exceeds θ. As to the impact of using lower threshold values, we will discuss it further in Section 5. In the end, a decision is made for every 330 ms in the continuous microphone input.

#### 4.2.2. Vaporizer Power Control

When the decision for an audio segment is Mosquito, the vaporizer power is turned on. It applies an active high signal to the load switch through the General Purpose Input Output (GPIO) of the MCU. The load switch reacts to the active high signal of the GPIO pin by supplying 5 V power to the vaporizer. Once powered on, it operates for a duration of $L_{power}$, which we set to one hour in the current prototype.

The reason is based on of Buhagiar [23] that pyrethroid-type metofluthrin (insecticidal component) spreads rapidly in the air, and no biting activity was observed in mosquitoes up to 5 m away from the emitter after 10 min of metofluthrin exposure. We assumed that maintaining the power supply for only one hour in the proposed system would be sufficient to kill any mosquitoes in the room. When the power of the electric mosquito repellent is turned off, the mosquito detection system again proceeds with the mosquito sound detection operation.

The power control operation is illustrated in Algorithm 1. The accumulation and averaging of classification results (Section 4.2.1) are in lines 7–12. If the average score of the Mosquito class C.Mos exceeds a detection threshold θ, the power is turned on for the vaporizer (line 23). However, additional processing occurs to suppress false positives further (lines 15–22). The details of the false positive suppression logic will be detailed in Section 5.4; however, the core idea is that there should be at least $X_{fp}+1$ segments for which the classification was Mosquito in a time window of $L_{win}$ milliseconds. The heuristic comes from our observation in in situ tests through which we find the appropriate parameter values. We will revisit Algorithm 1 in Section 5.4. Once the vaporizer begins operation, it turns off after $L_{power}$ (lines 28–32).

### 4.3. Deploying the Tinyml Application on Target Platform

In this section, we describe how the TinyML application discussed in Section 4.2 is deployed on the hardware discussed in Section 4.1.

Converting a trained Tensorflow model to run on a MCU that use C++ language requires a two-step process [24]. First, we convert a Tensorflow model stored as a flat buffer in serialized format into a Tensorflow lite model via the Tensorflow lite converter. In this paper, two scripts provided by Google are used to convert the Tensorflow lite model. This script records the weights and biases of checkpoints once every 100 steps and creates a static graph of the logged checkpoints with updated weights. The graph is written to a file called tiny_msqt.pb. Files can be converted to Tensorflow lite using TOCO, Tensorflow lite’s command-line interface. When TOCO runs, the tiny_msqt.pb file is converted to a tiny_msqt.tflite file containing the TensorFlow Lite model.
**Algorithm 1** Power control operation.1:$T_{now}\leftarrow 0$                                     ▹ is the current time2:$T_{mos}\leftarrow 0$                    ▹ is the last time mosquito sound was positively classified3:$T_{power}\leftarrow 0$                            ▹ is last time vaporizer was powered on4:$I_{mos}\leftarrow FALSE$                      ▹ flags mosquito classification within last 10 s5:$I_{power}\leftarrow FALSE$                           ▹ indicates vaporizer power level6:**while** true **do**7:    // CNN classifier operates continuously8:    C←0                   ▹ is the accumulated scores vector (Silence, Noise, Mosquito)9:    **for** $1:MAX_{proc}$**do**                    ▹ can only process $MAX_{proc}$ slices per $L_{seg}$10:        $slice\leftarrow MIC(L_{slice})$                    ▹ obtains a 30ms sound slice from mic11:        C←C+cnn_classify(slice)                ▹ scores vector for the current slice12:    **end for**13:    // Power control check (ON)14:    **if** $C.Mos/MAX_{proc}>\theta$**then**                ▹ last segment contains mosquito sound15:        **if** $I_{mos}==FALSE$**then**                   ▹ first positive since last vaporization16:           $I_{mos}\leftarrow TRUE$; $T_{mos}\leftarrow T_{now}-L_{slice}$                   ▹ register the event17:        **else**18:           **if** $T_{now}-T_{mos}\leq X_{fp}\cdot L_{seg}$ **then**19:               Continue                     ▹ coalesce the first $X_{fp}$ positives (Section 5.4)20:           **else**21:               **if** $T_{now}-T_{mos}<L_{win}$**then**                    ▹$X_{fp}$+ positives in $L_{win}$22:                   $I_{mos}\leftarrow FALSE$; $I_{power}\leftarrow ON$; $T_{power}\leftarrow T_{now}$23:                   power_control(ON)                    ▹ turn on vaporizer for $L_{power}$24:               **end if**25:           **end if**26:        **end if**27:    **end if**28:    // Power control check (OFF)29:    **if** $I_{power}==ON$ & $T_{now}-T_{power}>L_{power}$ **then**30:        $I_{power}\leftarrow OFF$31:        power_control(OFF)                     ▹ vaporizer has been on for $L_{power}$32:    **end if**33:**end while**

Second, we convert the Tensorflow lite model to a C++ array. Many microcontroller platforms do not natively support file systems. Therefore, the easiest way to use a Tensorflow lite model in a program is to compile it after including the model as a C++ array [25]. Thus, using xxd command that creates a hex dump of a given file or standard input on Linux, tiny_msqut.tflite file is converted into a byte array in the C++ language so that it can be used in a TinyML application. The CNN model converted into a byte array is written into a tiny_msqt_model.cc file for use by the TinyML application, whose size is 6968 bytes.

**Figure 6:**
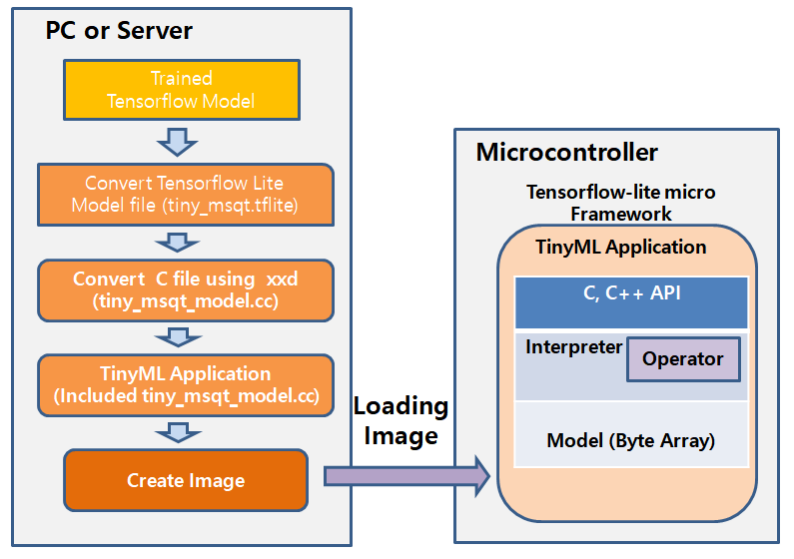
Deploying the mosquito detection TinyML application to MCU.

Note that a mosquito detection neural network defined as a byte array cannot do anything by itself. It is only part of the TinyML application. The TinyML application that contains the trained CNN model is transformed into binary image file [24]. The deployment is completed when the transformed image file is written to the MCU’s Flash memory. Flash writing is conducted through Universal asynchronous receiver/transmitter (UART) using a python code provided by Google [24]. A schematic of this deployed process is shown in Figure 6.

## 5. Prototype Evaluation

The intelligent mosquito repellent system that detects mosquito sounds and controls the vaporizer power was evaluated in a controlled environment. Note that experiments with live mosquitoes are nearly impossible to control. Therefore, the evaluation was not conducted in real operation conditions but, instead, in a controlled environment where the mosquito sound was played from a test sound source, not from live mosquitoes. Below, we first describe the test sound data. Then, we present the performance results, classification accuracy and power consumption.

Finally, we discuss the test of the developed prototype in target operating environment, from which we learned how to set some system parameters. Note that the performance on the target platform can be slightly different from that on the training platform (PC). The reason is twofold. First, for the model development on the PC platform the mosquito data are pure noise-free audio clips that are directly loaded from memory.

The test data on the target platform, on the other hand, come from the microphone in real time, with various unpredictable noise superimposed. Second, to control the false positives so that the proposed system is practical, we impose additional criteria for a positive decision on the mosquito presence as we briefly mentioned in Algorithm 1.

### 5.1. Test Setup and Data

**Figure 7:**
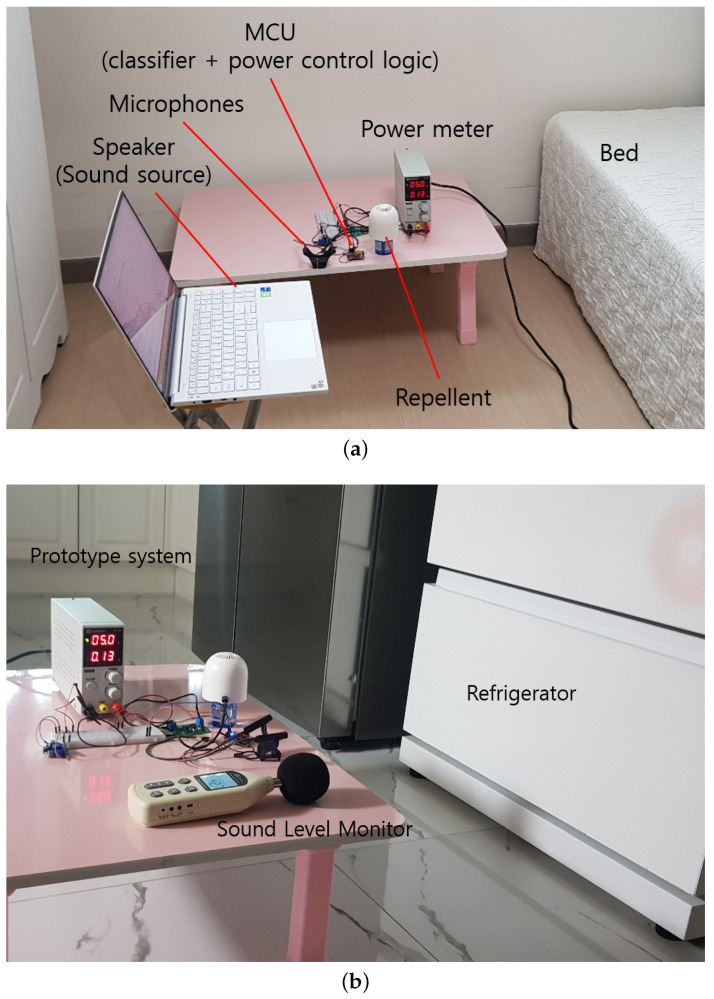
Test rooms and equipment configuration: (**a**) Bedroom (for false negative test). (**b**) Living room (for false positive test near refrigerator).

For the controlled tests, we used two rooms, a bedroom and a living room. The former is for the false negative test and the latter, for the false positive test. The setups are shown in Figure 7. The bedroom is 336 ×344 ×230 cm, and the volume is 26.58 m^3^. The living room is 370 ×750 ×230 cm, and the volume is 63.83 m^3^. The distance from the sound source to the smart device (i.e., its microphone) is varied by moving the table on which the sound source (speaker of a laptop) rests.

Two types of tests were performed to evaluate the performance of the prototype. The first is the false positive test, and the second is the false negative test. In order to measure the false positive rate of the developed prototype in the mosquito sound classification, we play various non-mosquito sound clips as shown in Table 4 (“Noise” in Table 5). They include the sound of a clock, rain, water, snoring and speech for the bedroom environment.

In the living room, we tested the sound of the refrigerator, dishwasher and the sound of water. Note that the false positive testing is performed with only the ambient living noises without playing the mosquito sound clips. These are natural sound sources, and thus their volumes are not controllable. The table shows the volumes for different non-mosquito noises as measured from the distance where the prototype system was located.

For the false negative test, we again utilize the humbugdb mosquito sound data (“Mosquito” in Table 5). Unlike the PC training, the prototype testing uses longer mosquito sound clips, i.e., 6 and 10 s clips. We assume these durations are given by the mosquito flying times within the sensing range of the microphone. In each Humbugdb sound clip, only a single mosquito is represented as we use those with the plurality type marked as ‘single.’ Note that there are no data for training and validation because they have been completed on the PC platform. Mosquito sound volume was set to 30–33 dB [26], as measured by a Digital Sound Level Meter [27] at a distance of 9 cm from the speaker. The distance from the sound source to the microphone was increased by 10 cm from 10 cm up to 140 cm. While the test was performed, there was natural indoor living and background noise present.

### 5.2. Accuracy

If the sensitivity of the mosquito detection neural network is low, the problem of false negatives occurs. In that case, the vaporizer will not be turned on when there are mosquitoes. In contrast, if the system falsely identifies noise as the mosquito sound, the vaporizer will operate when there are no mosquitoes. We need to avoid both these possibilities.

However, the reason to buy mosquito repellent is to avoid mosquito bites even with some exposures to chemicals and energy use; therefore, the true negative problem is considered more serious for customers. Unfortunately, false positives and false negatives are in a trade-off relation. What we should do is to strike a balance between the two. To that aim, we perform two types of tests to understand the false positive rate (FPR) and the false negative rate (FNR) characteristics of the developed prototype system.

#### 5.2.1. False Positive Test

When the mosquito sound determination is made every 330 ms, Table 6 shows the FPR performance for the decision threshold of θ=210 for various non-mosquito sound types. Overall, the average FPR is approximately 0.2%.

Table 7 shows the average FPR as we vary θ. We notice that the average FPR grows unbearably large as we lower the detection threshold from 210. This is why we use θ=210 in our prototype. Still, the mean time to a false positive detection is less than 3 min. Given the vaporizer operation time $L_{power}$ set to one hour as in Algorithm 1, one can see that the vaporizer may continuously operate due to frequent false positives caused by the noises from everyday life. Consequently, the developed system would not achieve any chemical- or power-reducing effects.

We address this problem as discussed in Algorithm 1 by requiring at least $X_{fp}+1=3$ consecutive or $X_{fp}$ non-consecutive segments or to be positively classified as Mosquito in $L_{win}=10$ s. The chosen value of $X_{fp}$ is based on our in situ experiments that will be discussed in Section 5.4. Considering mosquito’s flying speed in the considered microphone sensing range we do not believe that this requirement is excessive; however, we will explore the impact of this FPR suppression logic on the FNR performance below.

#### 5.2.2. False Negative Test

**Figure 8:**
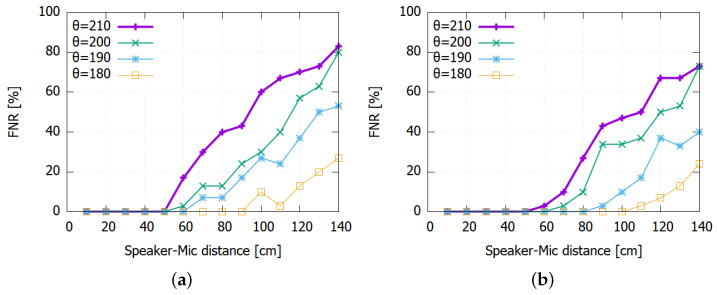
False negative rate for different decision thresholds (θ): (**a**) 6 s. (**b**) 10 s.

To measure the FNR, we use the mosquito sound clips with two different lengths (10 s and 6 s) in a closed room during day. The room was relatively silent; however, there was also casual background noise including clock ticking and outside noise. Figure 8 shows the test result when we vary the distances from the sound source to the microphone from 10 cm to 140 cm.

**Figure 9:**
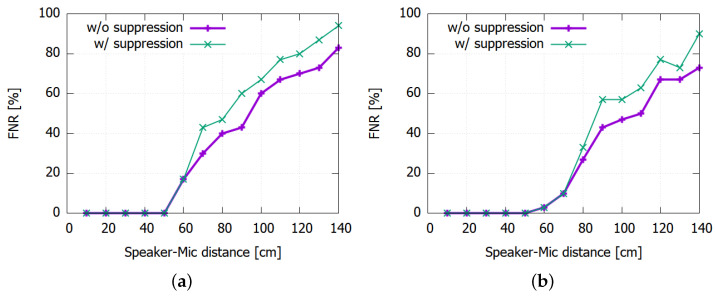
Detection rates at θ=210 with and without FP suppression: (**a**) 6 s. (**b**) 10 s.

Again, the FNR is also governed by the detection threshold θ where lower thresholds lead to lower FNR values. For instance, using a threshold as low as 180 makes the prototype detect the mosquito sound with low FNR at up to one meter. However, due to the higher FPR that would result from such low θ values, we set θ=210 in our prototype. For larger distances than 50 cm, however, the FNR can increase significantly in return. Moreover, applying the FPR suppression logic discussed in Section 5.2.1 can further aggravate the problem as shown in Figure 9.

**Figure 10:**
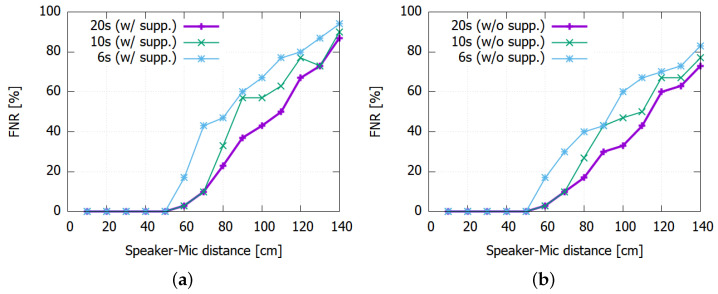
Impact of extending $L_{win}$ to 20 s: (**a**) With suppression. (**b**) Without suppression.

There can be at least two approaches to lowering the FNR. First, we could exploit the fact that the FNR is visibly lowered when the exposure to the mosquito sound is longer in Figure 9b. We could lengthen $L_{win}$ to make the system more patient and give more time to find more positively classified segment in $L_{win}$. For instance, Figure 10 shows the result for $L_{win}=20$ s. We can observe that the FNR is improved for both FPR-suppressed and non-FPR-suppressed cases. In particular, the suppressed case with $L_{win}=20$ s shows comparable performance with the non-suppressed case with $L_{win}=10$ s. It shows that forcing the system to use a longer time window $L_{win}$ can offset the FNR increase.

Another approach pertains to the hardware. Instead of using a general purpose microphone as in our prototype (with the passband of 200 to 4000 Hz), we could use one with a narrower frequency band targeted at the mosquito wingbeat. Specifically, a microphone that more tightly envelops the wingbeat frequency of female mosquitoes (passband of 400 to 500 Hz), would increase the sensitivity and, hence, lower the FNR.

### 5.3. Power Consumption

Our measurements of the prototype and the commercial product without the proposed enhancement show that the device dissipates 5.4 mA at 3.3 V when it performs only sound classification without operating the vaporizer. Thus, the power consumption is 17.69 mW. When the vaporizer is operating in the commercial system without the proposed enhancement, the current dissipation is 804 mA and the voltage is 5 V. It amounts to a power consumption of 4020 mW. Based on the measurement results, Table 8 estimates the energy consumption of the proposed system as a function of the vaporization hours.

Compared to the commercial system that continuously consumes 4020 mW, the commercial repellent uses 227 times more power in the absence of mosquitoes. For a vaporizing hours *k*, the energy consumption reduction factor is approximately 8/k. Note that when the vaporizer is on, the detection logic can be turned off. Furthermore, note that once a mosquito is detected, the vaporizer operates at least $L_{power}=1$ h; therefore, there is no operation time between 0 and 1 h.

**Figure 11:**
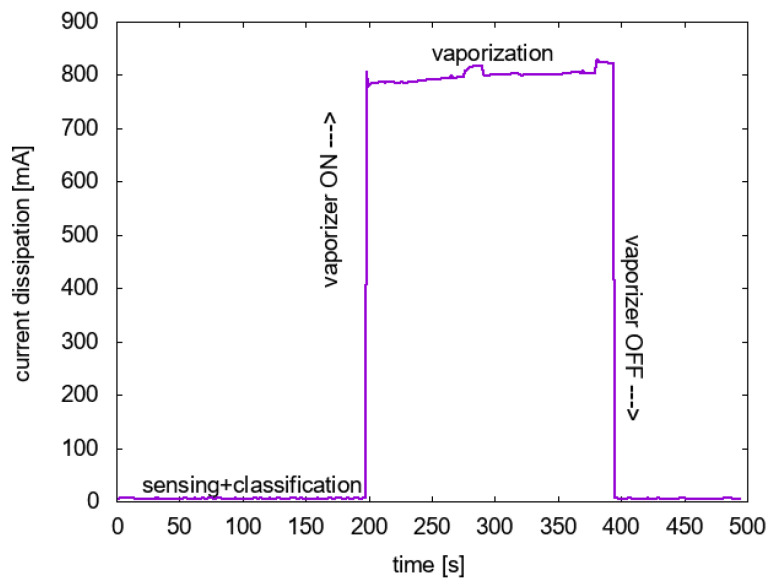
Current dissipation comparison between sensing + classification and vaporization.

Through actual measurements on the prototype, Figure 11 compares the current dissipation when the vaporizer is on and when off. In this measurement operation, the vaporizer is turned on for at approximately 200 s, before and after which only the sensing and classification operation is performed. Although not shown in the figure, the voltage applied to the sensing and classification is also lower. Therefore, the energy consumption when only the sensing and classification is on is orders of magnitudes lower than when the vaporizer is on. The benefit from accurate mosquito sound detection is well justified from the perspective of energy saving, not to mention the reduction of the amount of vaporized chemical.

### 5.4. In Situ Test in a Controlled Environment

Finally, we present the results of in situ testing of the prototype in a partially controlled environment. Using live mosquitoes in the tests was infeasible; thus, the mosquito sound clips were used instead. Therefore, the test results should only be interpreted as the potential of the proposed idea, not definite proof due to the sound source control. All other environmental factors were more natural. As the electric liquid repellent device will mostly be used in bedrooms during night, we monitored the performance of the prototype in a bedroom for one week.

**Figure 12:**
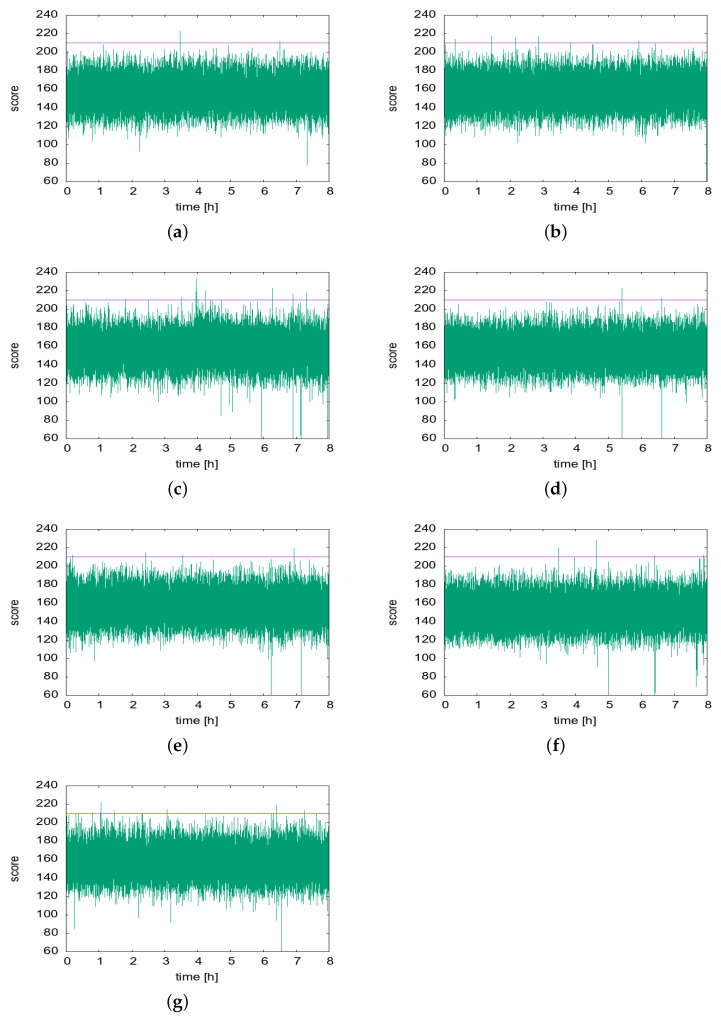
Daily “Mosquito” scores: (**a**–**g**), respectively, correspond to 24 May 2022 through 30 May 2022.

Figure 12 shows the scores of the Mosquito class, recorded for each 330 ms segment for eight hours (23:00 h–07:00 h) each day for one week from 24 May through 30 May 2022. Note that no artificial mosquito sound was played for this experiment. Therefore, the number of “Mosquito Decisions” shown in the table (It is practically impossible to directly confirm the matching presence of mosquitoes for positively classified segments. However, on two days, 26 and 29 May, mosquitoes were found in the morning after the experiment hours.). The results show that every day there were a few occasions where the threshold θ=210 was exceeded.

Table 9 partitions the positive classification events according to the number of consecutive segments in the positive classifications. In the table, we notice that out of the total 43 positively identified segments, most are isolated (single) occurrences or two consecutive occurrences. However, it is less likely that the mosquito sound is detected for only less than a second (330 ms or even 660 ms) in real life. Therefore, if we regard these two most frequent cases as the positive classification of a single segment.

As a consequence, the false positives can be significantly reduced from 43 occurrences (6.1 h per day on average) to at most eight occurrences (1.1 h per day on average) (However, mosquitoes were found on May 26 and 29; therefore, the two hours of vaporizer operation were not in vain. If this circumstance is accounted for, at most, six occurrences of vaporizer operations were the result of false positive detection, reducing the unnecessary operation to less than an hour a day.).

Note that the former would not justify the complexity cost of the developed system because only two hours of unnecessary operation will be suppressed each day. In the latter, however, an hour could be much more tolerable than up to eight hours of unnecessary operation as in the currently sold products. This is the motivation behind our decision to include the false positive suppression in Algorithm 1 in lines 13–27, where we set $X_{fp}=2$ segments. However, even if $X_{fp}=2$ segments are positively classified within $L_{win}$ but not consecutively, we assign a positive decision because positive classifications recurred over a longer period than 660 ms.

With these false positives suppression, the number of final decisions to turn on the vaporizer is significantly decreased. Table 9 shows that eight such decisions were made over a week. Note that the reason that the number of FP-suppressed decisions on 29 May is not 3 but 2 is because there were four consecutive positive segments followed by three consecutive positive segments within $L_{win}=10$ s. Therefore, it triggered the vaporizer only once. Furthermore, there were no instances of one or two positively identified segments that satisfy our criteria for vaporization that there should be either more than three positively classified segments or two or more non-contiguous segments within $L_{win}$.

## 6. Conclusions

A neural network model that detects mosquito sounds was developed using TinyML, and we demonstrated how it can be embedded in a commercial electric liquid mosquito repellent at a modest cost. Through training over mosquito sound data, the neural network model achieved high accuracy in detecting mosquito sounds in close proximity of the prototype device. By linking the detection model to the vaporization control in the device, the measured energy consumption was significantly reduced even when the detection model continuously classified the sound input.

This also contributes to the reduction of the chemical unnecessarily vaporized and subsequently inhaled by human users. In contrast, current commercial products operate continuously, consuming high power and exposing the user to the chemical during the entire operating time of the device. We believe that the developed prototype in this paper presents a good evidence that many everyday devices can be enhanced to have intelligence through TinyML and can reap substantial benefits.

## Figures and Tables

**Table 1:** Data set configuration for on-PC model development of the neural network that classified mosquito sounds.

Data Set	Total # of Clips	# of Mosquito Clips	# of Noise Clips	
			Living	Silence (Google)
Training	1370	806	282	282
Validation	165	97	34	34
Test	165	97	34	34
Total	1700	1000	350	350

**Table 2:** Model parameters.

Layer	Tensor Size (Dimension)	# of Weights	# of Biases	# of Params
Input	49 × 40 × 1	0	0	0
2D Convolution-1 (C1)	45 × 36 × 8	160	8	168
Subsampling-1 (S1)	23 × 18 × 8	0	0	0
2D Convolution-2 (C2)	19 × 15 × 8	1280	8	1288
Subsampling-2 (S2)	10 × 8 × 8	0	0	0
2D Convolution-3 (C3)	6 × 5 × 8	1280	8	1288
Fully Connected (FC)	64 × 1	15,360	64	15,424
Output	3 × 1	192	3	195
Total		18,272	91	18,363

**Table 3:** Training environment.

Environment	Configuration
OS	Ubuntu 18.04
GPU	NVIDIA GTX1050, CUDA 10.1, cuDNN 7.6.5
Build Tool	Bazel Release 0.26.1
Deep Learning Framework	Tensorflow 1.15.4
Programming Language	Python 3.6.9

**Table 4:** Non-mosquito sound clips for the false positive test; “Silence”: mixed sounds from outside and silence in room.

Location	Sound	Dist. (cm)	Vol. (dB)
Bedroom	Silence	-	30–35
	Clock	130	30–40
	Conversation	120	45–65
	Snoring	120	45–65
Living Rm.	Sizzling Pan	150	55–75
	Refrigerator	60	35–45
	Water	180	55–70
	Dish Washer	230	50–60

**Table 5:** Data set to test the target platform to classify Mosquito sound and noise.

Location	Mosquito	Duration (s)	Dist. (cm)	Vol. (dB)
Bedroom	20	6	10–140	30–33
	20	10		
Total	40	-	-	-

**Table 6:** False positive test results.

Location	Sound	#Errs./#Tests	FPR (%)
Bedroom	Silence	6/2400	0.25
	Clock	3/1200	0.25
	Conversation	3/1200	0.25
	Snoring	2/1200	0.17
Living Rm.	Sizzling Pan	3/1200	0.25
	Refrigerator	2/1200	0.17
	Water	2/1200	0.17
	Dish Washer	1/1200	0.08

**Table 7:** False positive rate as a function of classification threshold.

θ	Average FPR	Mean Time to False Positive (s)
210	0.2%	166
200	0.3%	101
190	1.0%	33
180	3.9%	8

**Table 8:** Energy consumption of the proposed system.

Vaporizing Hours	Energy Consumption	Reduction Factor
0	17.69 mW × 8 h = 141.5 mWh	227
1≤k≤8	4020 mW ×k h + 17.69 mW ×(8−k) h	≈8/k

**Table 9:** Positively classified segments and final decisions to turn on the vaporizer during in situ testing.

Consecutive Segments	5/24	5/25	5/26	5/27	5/28	5/29	5/30	Total
1	-	3	6	2	3	2	4	20
2	1	3	2	2	2	-	4	14
3	1	-	1	1	-	2	-	5
4	-	-	-	-	-	1	2	3
5	-	-	1	-	-	-	-	1
Non-suppressed	2	6	10	5	5	5	10	43
FP Suppressed Decisions	1	0	2	1	0	2	2	8

## Data Availability

Not applicable.
